# Histochemical and biochemical analysis of collagen content in formalin-fixed, paraffin embedded colonic samples

**DOI:** 10.1016/j.mex.2023.102416

**Published:** 2023-10-08

**Authors:** Nicholas Baidoo, Gareth J. Sanger, Abi Belai

**Affiliations:** a University of Roehampton, School of Life Sciences. Holybourne Ave, London. SW15 4JD, UK; b Blizard Institute, Faculty of Medicine and Dentistry, Queen Mary University of London, London, UK

**Keywords:** Collagen, Colon, Masson's Trichrome, Picrosirius red, Hydroxyproline, ImageJ, Human, Analysis of collagen content in formalin-fixed, paraffin-embedded tissues.

## Abstract

Collagen is the most abundant structural protein and extracellular matrix component in mammals. In the colon, collagen fibres reside in all the major sublayers; namely, the mucosa, submucosa, muscularis externa and the serosa. Methods to quantify collagen content in formalin-fixed, paraffin-embedded (FFPE) stained sections are required and image analysis offers a technique by which the spatial distribution and localisation of collagen fibres can be easily measured. This laboratory protocol was developed from established techniques using FFPE colon. Human colonic samples embedded transversally in paraffin wax were serially sectioned and stained with either Masson's trichrome (MT) or Picrosirius red (PSR). Quantitation estimation of collagen content in each sublayer was performed via ImageJ processing. Hydroxyproline content was quantified using a rapid and sensitive assay in sectioned tissue. Either MT or PSR staining followed by morphometric image analysis via ImageJ provided equally appreciable quantitative results. Moreso, analysis of hydroxyproline content in our samples indicate that this protocol could be useful in retrospective studies for FFPE samples. This laboratory protocol provides a systematic and reproducible method that can be utilized to accurately assess collagen content in individual sublayers of the colonic wall as well as detection of overall hydroxyproline content in FFPE specimens.

## Graphical abstract

**Figure:**
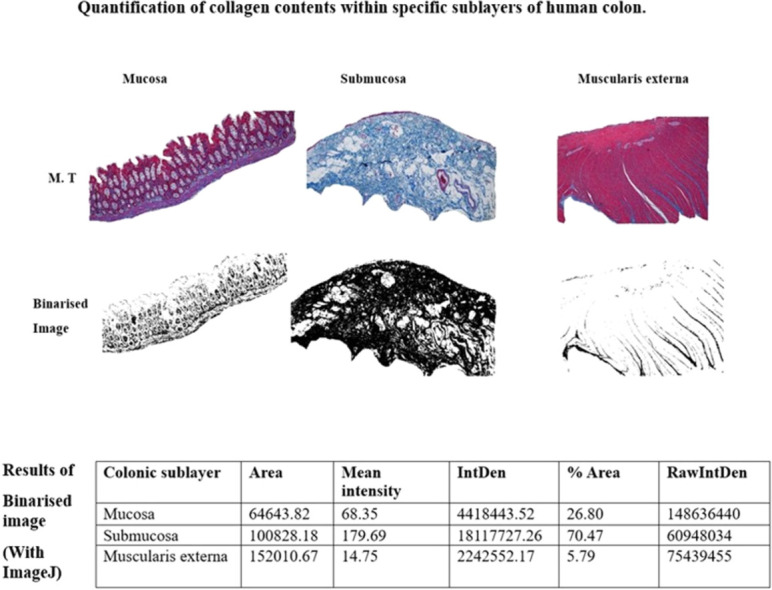
Quantification of total collagen content within sublayers of human colon

Specifications table

**Table:** 

Subject area:	Agricultural and Biological Sciences
More specific subject area:	Histology
Name of your protocol:	Histochemical and biochemical analysis of collagen in FFPE human colon
Reagents/tools:	Rotary microtome, Microtome blade, Water bath, Forceps, Paintbrush, Microscope glass slides, Lab coat and gloves. Xylene, Industrial methylated spirit (IMS), Harris haematoxylin, 0.5% acid alcohol (0.5ml of concentrated HCl + 99.5ml of IMS), Eosin, Scott's tap water, Cover glass, ArtisanLink autostainer (Sakura, Tokyo, Japan), PSR solution (0.1% of Sirius red in saturated aqueous picric acid), acidified water (0.5% acetic acid), Pertex or mounting medium. Brightfield microscope, ImageJ processing software. Total Collagen Assay kit (QuickZyme Biosciences, Netherlands), 12M, 6M and 4M HCl, Screw-capped sterilized tubes, Adhesive plate seals, Single and/or multichannel pipettes, Eppendorf centrifuge, Incubator or thermoblock for heating at 95^0^ C or 65^0^ C, Microplate reader capable of measuring at wavelength of 570nm, Histoclear/xylene, Microplate shaker and glass tubes
Experimental design:	Macroscopically normal human colon was obtained following surgery for non-obstructed bowel cancer, after informed written consent. The sections of colon were obtained at least 5-10 cm away from the tumor. Patient records were examined for current medication and comorbidity. Tissues (∼ 10 × 10 mm cut) were routinely fixed in 10% neutral buffered formalin and processed in alcohol, xylene and paraffin wax overnight. Colonic specimens were paraffin-embedded transversally to demonstrate mucosal, submucosal, muscularis externa and serosal layers in an embedding mold. Slides were assigned codes during microtomy to conceal the age of the patients. Carefully consistent serial-sections at 4-µm-thickness using rotary microtome were generated. A total of 50 serial sections per sample were cut and up to a depth of about 200 µm into the colon was used to evaluate total collagen content. Histochemical staining was employed followed by a reproducible step-by-step morphometric image analysis via ImageJ. Hydroxyproline content was quantified using a rapid and sensitive assay in sectioned colonic tissue.
Trial registration:	N/A
Ethics:	**Patient consent:** All patients provided written informed consent for the donation of tissue (REC 10/H0703/71; East London ethics committee). **Ethics approval:** Approved by the University of Roehampton (LSC 21/339) and the East London (REC 10/H0703/71) ethics committee.
Value of the Protocol:	• MT and PSR are cheap to buy, easy to access and is still currently in use for routine connective tissue morphometric analysis. The standardized approach utilized in microtomy, staining, imaging and analysis can be adopted to study collagen distribution in paraffin processed tissues. • The blue-stained structures for histochemical staining were maximally separated by the “Image Threshold” plugin in ImageJ and a standardisation step enabled a determination of the total collagen content within the functional layers of the human colon. • The protocol provides a systematic and reproducible method that can be utilized to accurately assess collagen content localizations in individual functional sublayers of the colonic wall as well as detect overall hydroxyproline content in FFPE specimens. The sensitivity of this protocol is such that it is now applicable to quantify hydroxyproline content in FFPE samples using only a few 10µm sections.

## Description of protocol

### Background

Collagen is the most abundant structural protein and extracellular matrix (ECM) component, with over 28 different subtypes found in mammals [1]. The colon wall consists of four anatomically distinct functional layers namely: mucosa, submucosa, muscularis externa and serosa. In general, collagen fibres are present in all the sublayers of the colonic wall providing structural support to resident cells. Thus, the propulsive and segmental movements of the colon, in addition to its role in the temporary storage of luminal content for further absorption of water and salts prior to defecation, requires a biomechanical contribution from the submucosa and muscularis externa [2,3].

It is important to understand the normal distribution of collagen within the sublayers of the colon, and how this may change in some collagen related disorders. These include those associated with tissue damage, such as inflammatory bowel disease [4] diverticular disease and anastomotic leakage after large bowel surgery [5]. In addition, the human colon, at the level of submucosa and muscularis externa, shows an increase in total collagen fibres among the elderly [6,7] indicating that the functions of these sublayers may be vulnerable to age-associated physiological changes.

Assessment of the amount of collagen fibres as an index to evaluate collagen fibres within histological sections, employs various tinctorial stains and techniques of quantification. Among these, Masson's trichrome (MT) and Picrosirus red (PSR) staining (both dyes do not distinguish various collagen family but identify almost all the collagen types) are routinely used for demonstration of colonic collagen fibres in both diagnostic and research settings [8]. However, analysis of collagen fibre contents in individual functional sublayers are often challenging. This is because histological staining often utilises more than one dye which often co-localize in the same area within the colon wall and individual sublayers have to be separately analysed. A solution for these challenges may be found in an image processing programme ImageJ, which has the capacity to separate colours into 8-bit monochromatic colour thus aiding accurate quantification [9,10]. In addition to assessing localisation of collagen fibres in histological section, the measurement of hydroxyproline concentration is considered a gold standard technique to quantify total collagen in tissue samples. Hydroxyproline amino acid is exclusively present in all collagen samples [11]; therefore, by quantifying the amount of hydroxyproline content as a fixed percentage in tissue, collagen content can be indirectly deduced. The use of fresh human colonic tissue which is more difficult to obtain, compared to formalin-fixed paraffin-embedded (FFPE) tissue (the major way histological samples are stored), has always been a choice for most collagen extraction investigations. Nevertheless, a satisfactory analysis of hydroxyproline content in FFPE tissues obtained from other parts of the body has been described [12], and a simple and sensitive assay for accurately quantifying collagen content in a FFPE sample by this method is now available.

Therefore, the aim of this protocol is to: (1) describe a convenient way in assessing collagen fibres distribution in the individual colonic sublayers with colour thresholding technique in ImageJ software and (2) evaluate hydroxyproline content using a spectrophotometric based assay in FFPE samples. The methods described herein were used to assess the pattern of distribution of total collagen contents in ageing human colon [6,7].

## MATERIALS AND METHODS

### Sample preparation and microtomy

Macroscopically normal colon tissue was obtained following surgery for non-obstructed bowel cancer, after informed written consent. The sections of colon were obtained at least 5-10 cm away from the tumour and were prospectively collected. Patient records were examined for current medication and comorbidity. None of the patients underwent surgery had any previous chemoradiotherapy or diagnosis of active inflammatory colonic disease such as diverticulitis known to affect collagen structure and organisation [13]. The samples (∼ 10 × 10 mm cut) were fixed, processed, embedded transversally and serially sectioned. The study was approved by the University of Roehampton (LSC 21/339) and East London Ethics Committees (REC 10/H0703/71).

#### Materials 1

Rotary microtome, Microtome blade, Water bath, Forceps, Paintbrush, Microscope glass slides, Lab coat and gloves.

• Carefully consistent serial-sections at 4-µm-thickness using rotary microtome (Leica Biosystems, Buffalo Grove, United States) were generated and mounted on superfrost-plus glass slides.
• A total of at least 50 serial sections per sample were cut and up to a depth of about 200 µm into the colonic biopsy was used to evaluate total collagen content. Two sections per slide at 16 µm separation depending on the size of the tissue.
• The first section of each sample/patient was used for routine haematoxylin and eosin (H&E) staining to exclude any tumour or active inflammation known to affect collagen structure [13].
• Histochemical staining analysis was performed at every fifth section (with a total of at least eight sections per patient). Before staining was performed, sections were deparaffinized in xylene, rehydrated and stained for routine H&E or Masson's trichrome or Picrosirus red.

### Histological staining

#### Materials 2

Xylene, Industrial methylated spirit (IMS), Harris haematoxylin, 0.5% acid alcohol (0.5ml of concentrated HCl + 99.5ml of IMS), Eosin, Scott's tap water, Cover glass, ArtisanLink autostainer (Sakura, Tokyo, Japan), PSR solution (0.1% of Sirius red in saturated aqueous picric acid), acidified water (0.5% acetic acid), Pertex or mounting medium.

#### Haematoxylin and Eosin staining

• Colonic tissue sections were manually stained in Harris haematoxylin, differentiated in 0.5% acid-alcohol and counterstained with eosin [14].
• The sections were then dehydrated in graded series of alcohol and cleared in xylene. Stained sections were then mounted with Pertex (Sakura) and coverslipped with glass slide (Sakura, Tokyo-Japan).
• Nuclei were stained dark blue and the cytoplasmic regions stained red to pink or orange colouration.

#### Masson's trichrome

• All sections were loaded on ArtisanLink auto stainer (Sakura, Tokyo-Japan) for Masson's trichrome staining.
• Stained sections were then mounted with Pertex and coverslipped with glass slide (Sakura, Tokyo-Japan).
• Stained components of the human colon sections yielded the following results: nuclei were stained black; cytoplasm, muscles and erythrocytes were stained red, collagen fibres appeared blue.
• In the absence of auto-stainer, manual staining can be performed [15].

#### Picrosirus red

• Sections were stained in PSR solution (0.1% of Sirius red in saturated aqueous picric acid) for 1hr [16].
• The sections were washed in two changes of acidified water (0.5% acetic acid) for 2min each, air dried and dehydrated in three changes of 100% ethanol.
• Stained sections were then cleared in histoclear and mounted with Pertex and coverslipped with glass slide (Sakura, Tokyo-Japan).
• In bright-field microscopy, collagen fibres appeared red on a pale-yellow background.

### Microscopy and Image analysis

#### Materials

Brightfield microscope, ImageJ processing software.

#### Image acquisition

• Sequential image capturing from mucosa, submucosa and muscularis externa was used to ensure that the entire stained sections were represented and that a minimum of at least 90 percent of the acquired images were analyzed.
• All images from colonic sections stained with histochemical staining were acquired with a 4X objective lens (numerical aperture = 0.20; white LED illumination light; Nikon instruments, Melville, NY) on an Eclipse C*i* brightfield microscope with DS-L4 camera (Nikon) under identical conditions.
• Standardization of imaging to maintain image-to-image consistency and if tonal linearity was used, this should be applied to all the samples.
• To maintain image integrity and clarity, all acquired digital images were stored in an uncompressed tagged image file format (TIFF) with 24-bit RGB; 23MB and 20.00 × 14.22 inches (2880 × 2048) resolution.
• Other best lossless image formats that can be edited or compressed without impacting image quality suitable for image analysis include TIFF with LZW (Lempel-Ziv-Welch) or ZIP and also PNG (Portable Network Graphic).

### Image analysis

#### Procedure for quantifying total collagen contents

• The procedure described herein for accurate quantification of total collagen content was performed with a Java-based image processing programme developed at the National Institute of Health (NIH), version 1.53g [9,10].
• There are five steps required for quantitating collagen fibres localizations in functional sublayers in colonic wall within histological section: installation of the ImageJ software and plugin, setting the scale or spatial calibration, separation of colour images by “Colour Threshold,” binarization of image, circumscribing the individual region of interest (mucosa, submucosa and muscularis externa) and measurement of positive pixel.
• Total collagen content for each layer of the colon was defined as the proportion of positive pixels or gray values within the mucosa, submucosal and muscularis externa area. Herein quantification of collagen contents in the colonic section based on MT staining were described.

#### Setting of scale

**Fig. 1:**
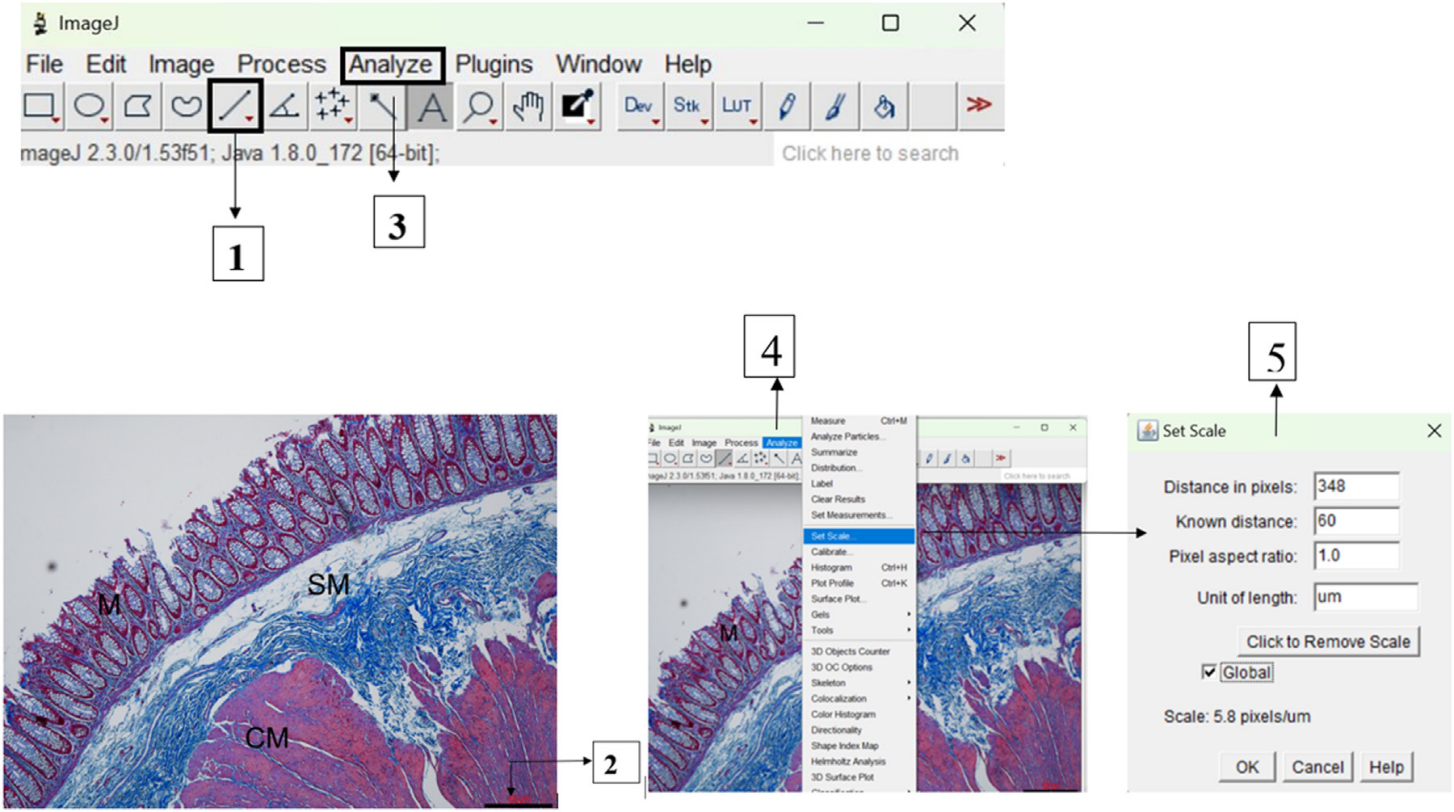
Method for analyzing Masson's trichrome stained colonic samples images with ImageJ programme. The image is first opened and Scale setting performed by **1**) The “Straight” line tool is selected and a straight line is drawn on the image as shown on **2.** On the “Analyze” menu (**3**), the “Set Scale” (**4**) automatically calibrates “Distance in pixels”, the length of the scale bar of 60 is entered into “Known Distance” box, and the unit in µm was then input in the “Unit of length” box. And when “Global” is checked, all the images for analysis were automatically scaled to 5.8 pixels/ µm. M: mucosa; SM: submucosa; CM: circular muscle; scale bar: 60 µm.

All images captured were taken at a known distance of 60µm and were all calibrated to provide a pixel-to-real-distance conversion factor. To do this, a straight line was drawn on the scale bar of the opened image using ‘straight line’ toolbar. As shown in Fig. 1 in the “Analyze” menu, “Set Scale” option was selected to automatically generate a dialogue box, so “Distance in pixels” as based on the scale bar, automatically calculates the distance in pixels as 348 and a “Known distance” of 60 is entered; “Pixel aspect ratio” set to 1.0 and “Unit of length” in µm. In this protocol, a calibrated scale of 5.8 pixels/µm was used in all the image analysis as the dialogue box was set to “Global” which provided a resolution of 496.55 × 353.10μm.

#### Colour separation staining with “Colour Threshold” method

**Fig. 2:**
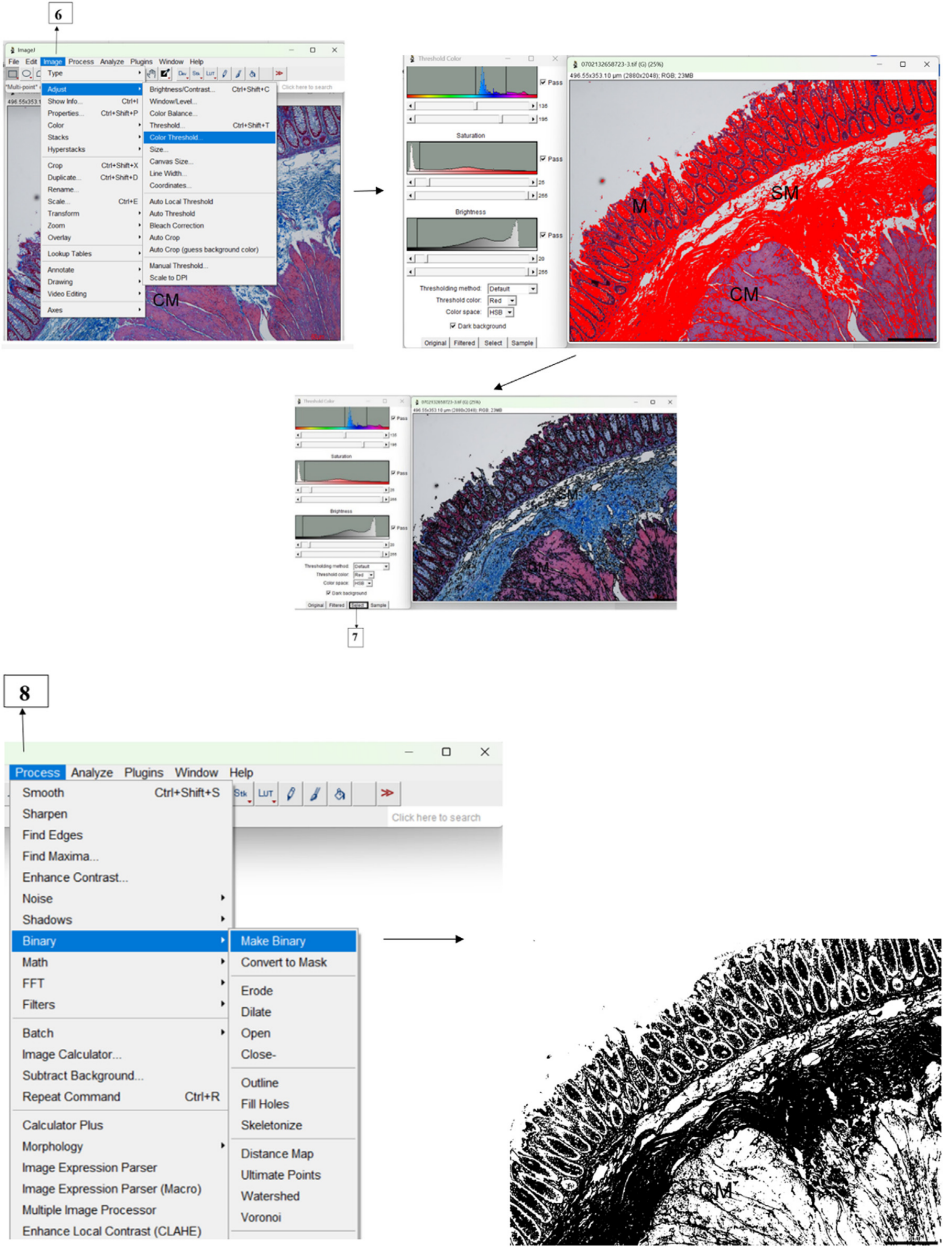
Separation and conversion of threshold images for quantification (**6**) Under “Image” menu, then to “Adjust” and when “Colour Threshold” is selected, a dialogue box appears. Manual threshold of Hue (135-195), Saturation (25-255) and Brightness (20-255) was set at these values and optimal blue colour intensity was achieved when ‘Selected’ (**7**). To convert the threshold image into 8-bit greyscale, (**8**) “Process” is selected from the menu, and when “Binary” and “Make Binary” is clicked, a greyscale image is achieved . Scale bar represent 60 µm.
• Manual thresholding values were set for MT staining: Hue (135-195), Saturation (25-255) and Brightness (20-255)
• A threshold colour dialogue box appears and by selecting “Default” thresholding method, Threshold Colour set to “Red” and Colour space to “HSB” (Hue, Saturation and Brightness). All colonic images were measured at the same HSB values as stated and when “Select” is clicked under “Threshold Colour” dialogue box, the threshold image is ready for binarization.
• The blue colouration representing collagen fibres in the colonic tissue was separated from background by manual colour threshold of Hue (H), Saturation (S) and Brightness (B) using the slider bars in ImageJ. In this experiment, the pixels within the threshold range are displayed in red. It is to note that each laboratory should establish their own blue-stained colour threshold values by calibrating HSB for differences between intensity of stain and microscopes. It is suggested that users can train their eyes on a simple control sample to achieve threshold values good enough for further analysis. As the human colonic submucosa is rich with collagen fibres, these were used as an internal control to determine and validate the HSB colour-space thresholds values. Herein, Hue values of 135-195 broadly select for only blue-stained colour within the colonic sample. By using the sliders, values for Saturation and Brightness were set to exclude or remove interfering background. For quantification purposes, it is important that these values should remain constant for all sections during image analysis to ensure consistent thresholding.

• As shown in Fig. 2, the selected calibrated colour image is converted into red and blue components representing the amount of collagen fibres in the specimen for binarization.

#### Binarization of images

• To convert the threshold image into 8-bit greyscale, “Process” is selected from the menu, then “Binary” and “Make Binary” is clicked, a greyscale image is achieved (Fig. 2).

### Analysis of collagen fibres in the sublayers of colonic wall

**Fig. 3:**
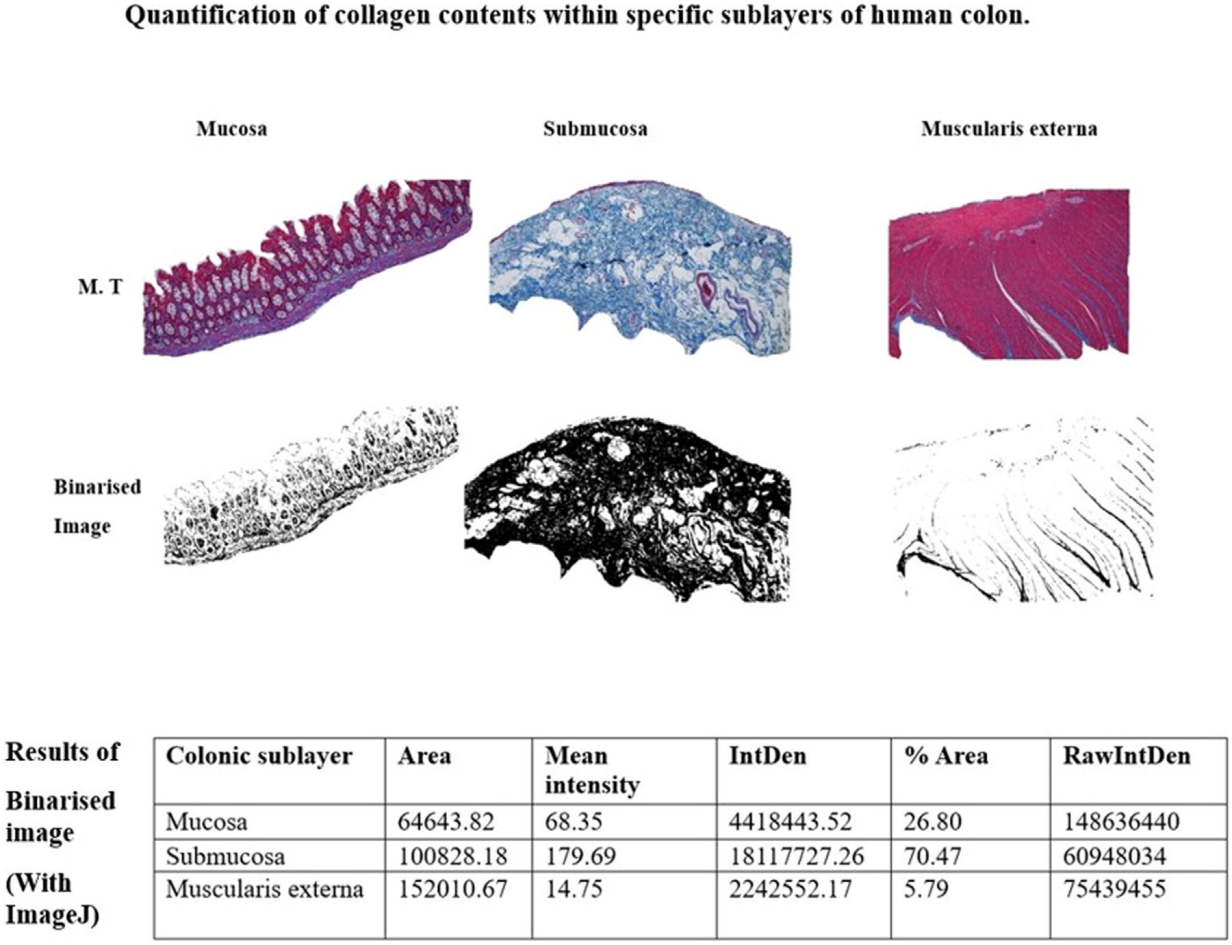
Quantification of total collagen fibres distributed in human colon using ImageJ software. Grayscale images of Masson's trichrome (MT) stained colonic sections were circumscribed with freehand tool in the mucosa, Submucosa and Muscularis externa. When “Analyze” from the menu and “Measure” is selected, the result of positive pixel per region of interest is presented. Scale bars represent 60 µm.

As described above and in Fig. 3, segmented 8-bit greyscale image can be used to quantify the amount of blue staining collagen fibres present in the selected regions of the colon for analysis. For the mucosal area, the “Freehand” drawing tool was selected and an area containing the epithelium, lamina propria and muscularis mucosae was drawn.

• Under the “Analyze” menu, “Tools” and “ROI Manager” was selected and when “Measure” is applied, the result is automatically presented.
• To avoid measuring a positive pixel representing collagen fibres more than once, the quantified area or region is erased by selecting “Edit” from the menu and when “Fill” is clicked, the analyzed area erased.
• Per analysis of mucosa layer, a similar method was applied to the submucosa (an area between the *muscularis mucosae* and the circular muscle layer).
• In respect to muscularis externa, a freehand line was drawn on an area comprising the circular and longitudinal muscle, with myenteric plexus embedded between the two muscle layers, and measured as described for mucosa and submucosa. Collagen analysis for serosal layer was not included in this protocol.
• For total collagen content within the *taenia coli* (TC), a tracing tool was manually selected to circumscribe an area around the edges of the *taenia coli* towards the serosa layer and around the myenteric plexus. A similar method was used to obtain an area of only circular muscle (CM) (an area around the edges of the smooth muscle towards the submucosa and the myenteric plexus without a ganglion). The total collagen content was automatically calculated from the positive pixels per the ROI [7].

## CRITICAL PARAMETERS

• Evaluation of at least 50 serial sections (16 µm separation) per sample were analyzed to ensure that at least up to 200 µm thickness of colonic samples was assessed. Collagen content differs within the sublayers and depth (levels within tissue section) in the same colonic sample. Assessment of collagen on just one thin section does not provide accurate quantitative outcome.
• All tinctorial staining were performed with the same batch of reagent under identical condition. Variability in the use of reagent may affect the staining outcome which subsequently can affect the colour spectrum during manual threshold.
• Either systematic image acquisition or scanning the entire stained slides is preferred. This is to ensure that at least 90% of the ROI per sample is analyzed to avoid randomly selected areas which can contribute to an element of bias.
• All histological images were taken under identical condition and ideally saved under uncompressed tagged image file format (TIFF) to maintain clarity and integrity.
• The amount of total collagen content within a tissue sample depends on the state of tissue (e.g., higher collagen content has been reported in diverticular samples [13], therefore it is crucial to assess the presence or absence of any active inflammation with initial Haematoxylin and Eosin staining.
• It is preferable to optimize threshold values and thresholding method (e.g., Default, Huang, Intermodes, IsoData) before any further quantification.

### Hydroxyproline analysis of collagen concentration

#### Materials

Total Collagen Assay kit (QuickZyme Biosciences, Netherlands), 12M, 6M and 4M HCl, Screw-capped sterilized tubes, Adhesive plate seals, Single and/or multichannel pipettes, Eppendorf centrifuge, Incubator or thermoblock for heating at 95^0^ C or 65^0^ C, Microplate reader capable of measuring at wavelength of 570nm, Histoclear/xylene, Microplate shaker and glass tubes.

#### Hydroxyproline assay

Hydroxyproline concentration in FFPE human colonic tissues was measured using an assay kit (*QuickZyme* Biosciences, Netherlands). This assay recognizes all types of collagen, irrespective of form (e.g., mature, immature, procollagen, degraded collagen, cross-linked collagen).

• Ten 10µm FFPE sections were weighed and transferred to screw-capped tubes. Samples were then divided into two groups. One group of samples were deparaffinised in histoclear (treated) and the other group without (untreated).
• Thereafter, 150 µl of 6M HCl was added and the samples hydrolysed for 20hr at 95⁰C in a thermoblock.
• Hydroxyproline standard solutions (6.25 – 300 µgmL^−1^) were prepared according to manufacturer's protocol. Briefly, the collagen standard was received as a stock of 1200 µg/ml in 0.02M acetic acid. 125 µl of the collagen standard is transferred to a screw-capped tube and mixed with an equal volume (125 µl) of 12 M HCl (final concentration 600 µg/ml in 6M HCl) and incubated in a thermoblock for 20hr at 95⁰C.
• After incubation, both the standard solution and sample solutions are cooled at room temperature, centrifuged for 10 min at 13,000 x g in an Eppendorf centrifuge. The supernatant was for further analysis.
• Further dilution required 200 µl of hydrolysate samples mixed with 100 µl of distilled water (samples are now in 4M HCl). Subsequent further dilutions were performed using 4M HCl.
• Both the standard concentration and sample solution were assayed in duplicate. 35 µl of standard solution and hydrolysate samples were pipetted into appropriate wells of the assay microplate and treated per manufacturer's instruction.
• Next, the plates were covered with an enclosed adhesive plate seal and incubated for 60 minutes at 60^0^ C in an oven.
• The plates were cooled on ice for a maximum of 5 minutes to room temperature. Before reading the absorbance, the plate was gently shaken.
• The plate absorbance was read at 570 nm on a multiskan ex microplate reader (Thermo Scientific, Singapore). The averaged blank readings were subtracted from the averaged duplicate readings for each standard (Table 1) and sample (Table 2).

**Table 1:** Collagen standard preparation

Standard label	Sample from	4M HCl	Distilled water	Conc (µg/ml)	Average absorbance reading (570 nm)
S1	125 µl hydrolysate stock	62.5 µl	62.5 µl	300	1.13
S2	120 µl S1	60 µl	-	200	0.81
S3	90 µl S2	90 µl	-	100	0.46
S4	90 µl S3	90 µl	-	50	0.25
S5	90 µl S4	90 µl	-	25	0.16
S6	90 µl S5	90 µl	-	12.5	0.10
S7	90 µl S6	90 µl	-	6.25	0.08
S8	0	90 µl	-	0	0.01

**Table 2:** Analysis of total hydroxyproline concentration in human ascending colon (Untreated; without histoclear and treated with histoclear)

Age (y)	Gender	Average Absorbance reading (570nm)-Untreated	Unknown samples (µg/ml) - Untreated	Average Absorbance reading (570nm).Treated	Unknown samples (µg/ml) - treated
43	Male	0.223	42.46	0.228	43.98
47	Male	0.179	30.51	0.183	31.43
51	Female	0.244	48.31	0.247	49.21
52	Male	0.178	29.91	0.179	30.32
53	Female	0.244	48.35	0.249	49.91
53	Male	0.174	28.99	0.177	29.76
53	Male	0.169	27.63	0.174	28.93
54	Male	0.186	32.35	0.192	34.02
55	Female	0.218	41.21	0.222	41.99
55	Female	0.196	34.93	0.198	35.65
56	Male	0.210	39.11	0.215	40.32
57	Female	0.181	30.81	0.182	31.19
59	Male	0.225	43.21	0.228	44.03
59	Male	0.168	27.19	0.172	28.32
60	Female	0.249	49.81	0.259	49.91
66	Male	0.245	48.76	0.251	48.91
68	Female	0.264	53.99	0.265	54.09
72	Female	0.281	58.69	0.283	59.10
74	Male	0.251	50.32	0.253	50.95
75	Male	0.281	58.69	0.284	59.55
77	Male	0.249	49.98	0.251	50.43
77	Female	0.279	58.19	0.282	58.89
79	Female	0.238	46.59	0.242	47.83
80	Female	0.216	40.58	0.219	41.34
82	Male	0.254	51.21	0.258	52.09
82	Male	0.277	57.49	0.280	58.11
84	Female	0.282	58.91	0.284	59.43
87	Male	0.208	38.31	0.217	40.93
88	Male	0.247	49.21	0.251	49.89
89	Female	0.218	41.23	0.223	42.56
90	Female	0.281	58.69	0.283	59.21

**Fig. 4:**
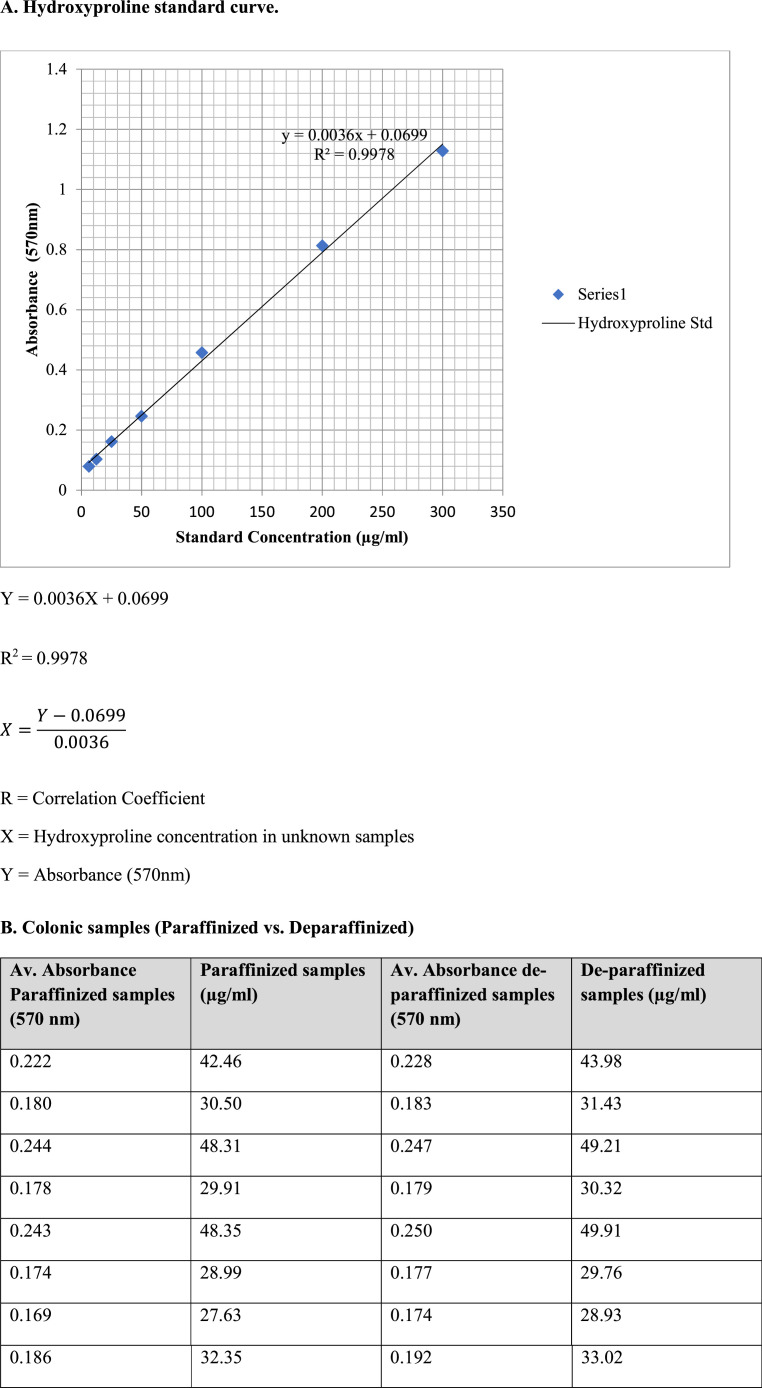
Representative examples of hydroxyproline content measurements in formalin fixed, paraffin-embedded colonic tissue. **(A)** graph demonstrate standard curve of mean absorbance (A_570_ – blank) against standard known collagen concentration. **(B)** The standard curve was used to convert the A_570_ values of the test samples (paraffinized vs. deparaffinized) to converted-hydroxyproline concentration of collagen in the hydrolyzed human colonic samples.

Human ascending colon were obtained from adult (< 65 years) and elderly (≥ 65 years) donors. Analysis of total hydroxyproline content measurements were performed using assay kit (*QuickZyme* Biosciences, Netherlands). One group of samples were deparaffinized in histoclear (treated) and the other group without (untreated). The plate absorbance was read at 570 nm on a multiskan ex microplate reader. A standard curve was prepared by plotting the mean absorbance (A_570_) of each standard against the collagen content concentration. The unknown concentrations of total hydroxyproline in treated and untreated colonic hydrolysates were deduced per volume of HCl used, based on the standard calibration regression curve.
• A standard curve was prepared by plotting the mean A_570_ (minus blank) of each standard on the y-axis against the collagen content concentration on the x-axis and applying a best-fit linearized curve through the points on the graph. The unknown concentrations of total hydroxyproline in treated and untreated colonic hydrolysates were deduced per volume of HCl used, based on the standard calibration regression curve (Fig. 4).

## CRITICAL PARAMETERS

• Colonic hydrolysate takes place at 95°C (not higher) for 20 hrs. Hydrolysis solution will evaporate if tubes are not tightly closed.
• The incubation time for colour development at 60°C must be done in an oven for 1 hr.
• If after hydrolysis of a colonic tissue a further dilution is required, the concentration should be multiplied with the dilution factor to give the hydroxyproline concentration in the hydrolyzed colonic samples.
• All sample size prior to processing were cut at the same dimension (i.e., ∼ 10 × 10 mm).

### Method Validation

**Fig. 5:**
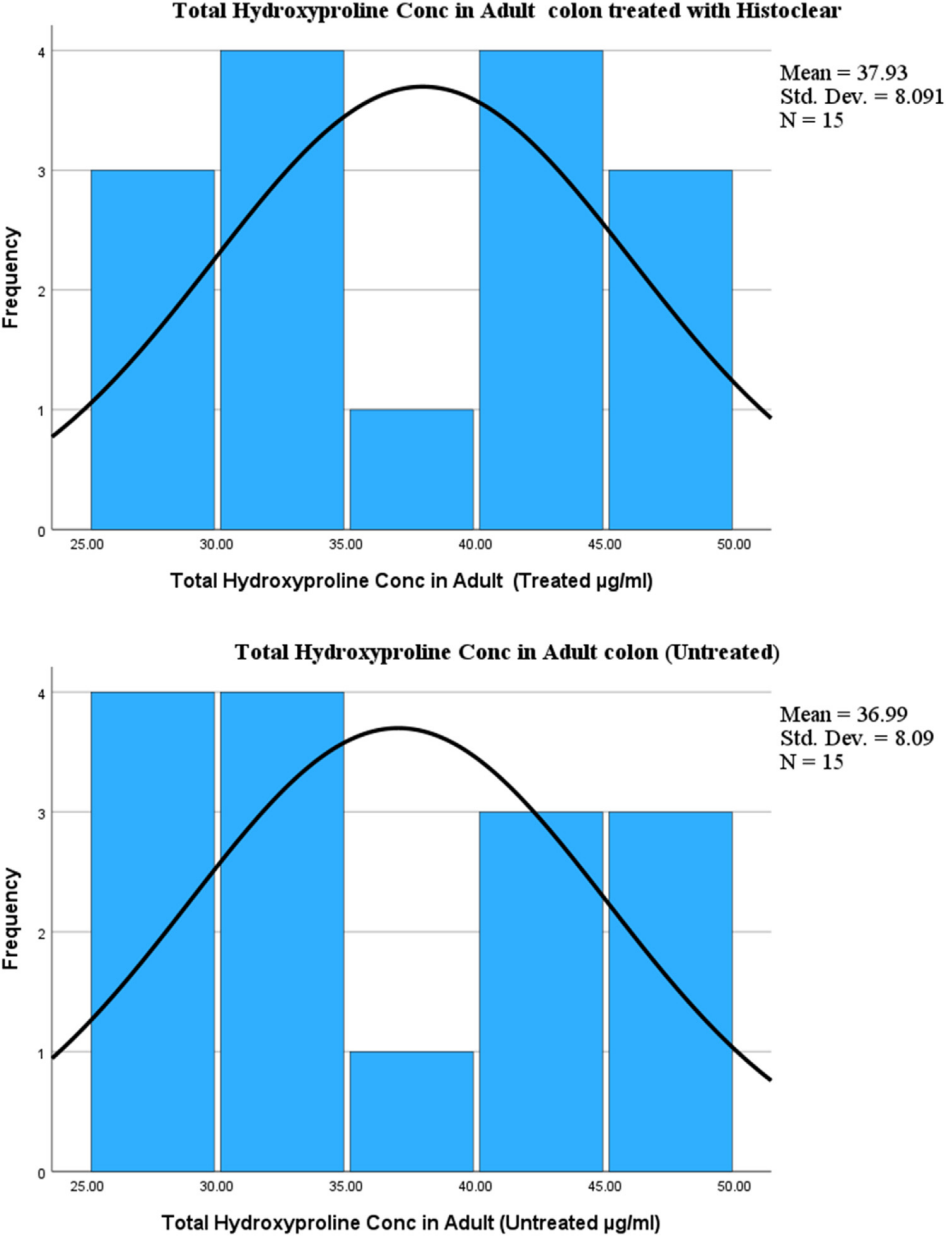
The histogram shows the distribution pattern of total hydroxyproline content in treated (with histoclear) and without treatment in formalin-fixed, paraffin-embedded adult colonic samples. The results from the mean result from treated (37.93; SD = 8.1) samples did not differ from untreated (Mean= 36.99; SD= 8.1).

• The sensitivity of this protocol is such that it is now applicable to quantify hydroxyproline content in FFPE samples using only a few 10µm sections.
• In the analysis of the amount of hydroxyproline content in colonic samples, previous studies reported no statistically significant differences in the amount of hydroxyproline content between treated (with histoclear) and untreated (with paraffin) samples [6,7], indicating that this technique can be employed without the use of xylene/histoclear (see Fig. 5).

## Results

**Table:** 

Tests of Normality						
	Kolmogorov – Smirnov^a^			Shapiro-Wilk		
	Statistic	df	Sig.	Statistic	df	Sig
Total Hydroxyproline Conc in Adult (Treated: µg/ml)	.189	15	.155	.881	15	0.49
Total Hydroxyproline Conc in Adult (Untreated: µg/ml)	.183	15	.187	.896	15	.081

a Lilliefors Significance Correction

The Kolmogorov-Smirnov test is non-significant (p > 0.05) for both total hydroxyproline concentration treated with histoclear and without treatment results, indicating that the data are normally distributed.

**Table:** 

Correlations			
		Total Hydroxyproline Conc in Adult (Treated: µg/ml)	Total Hydroxyproline Conc in Adult (Untreated: µg/ml)
Total Hydroxyproline Conc in Adult (Treated µg/ml)	Pearson Correlation	1	.998⁎⁎
	Sig. (2-tailed)		< .001
	N	15	15
Total Hydroxyproline Conc in Adult (Untreated: µg/ml)	Pearson Correlation	.998⁎⁎	1
	Sig. (2-tailed)	< .001	
	N	15	15

⁎⁎ Correlation is significant at the 0.01 level (2-tailed)

A Pearson correlation coefficient was computed to assess the linear relationship between the results obtained from total hydroxyproline concentration treated with histoclear and those without treatment in adult human colon. There was a positive correlation between the two variables, r = 0.998, n =15, p = 0.01, indicating that assessment of total hydroxyproline content in human colon can be achieved either with histoclear or without removal of paraffin-embedded wax.

## Conclusion

Using our protocol, collagen fibres within the sublayers of colonic samples stored as FFPE, as well as the total hydroxyproline contents, can be accurately assessed. Our protocol presents a simple method for the quantitative and objective measurement of the density of collagen fibres in MT- stained colonic samples using image analysis. Further, the use of QuickZyme collagen assay kit enabled detection of hydroxyproline contents in FFPE samples. The current approach to microtomy in this present study is regarded as the most effective method to generate comparable analysis as it is easy to repeat to obtain a consistent result. It is worth noting that collagen fibres content differs in various planes of the tissue; therefore, the use of just single section may not provide a robust result. The hydroxyproline content in colonic hydrolysates can be used as a direct evaluation of the amount of collagen present. In conclusion, this laboratory protocol provides a systematic and reproducible methods that can be utilized to accurately assess collagen content localizations in individual functional sublayers of the colonic wall as well as detection of overall hydroxyproline content in FFPE specimen.

## Ethics statements

**Patient consent:** All patients provided written informed consent for the donation of tissue (REC 10/H0703/71; East London ethics committee).

**Ethics approval:** Approved by the University of Roehampton (LSC 21/339) and the East London (REC 10/H0703/71) ethics committee.

## Funding

This research did not receive any specific grant from funding agencies in the public, commercial, or not-for-profit sectors.

## Acknowledgments

We thank the colorectal surgeons and pathologists of Barts and the London NHS Trust for providing tissue for laboratory use and all the technical staff at the School of Health Sciences, University of Roehampton for their immense effort in the organization of equipment for this project.

## CRediT authorship contribution statement

**Nicholas Baidoo:** Conceptualization, Methodology, Visualization, Investigation, Software, Validation, Data curation, Writing – original draft. **Gareth J. Sanger:** Methodology, Visualization, Validation, Supervision. **Abi Belai:** Validation, Writing – review & editing.

## Declaration of Competing Interest

The authors declare that they have no known competing financial interests or personal relationships that could have appeared to influence the work reported in this paper.

## Data Availability

Data will be made available on request.
